# Antiproliferative effects of D-allose associated with reduced cell division frequency in glioblastoma

**DOI:** 10.1038/s41598-023-46796-4

**Published:** 2023-11-09

**Authors:** Kenta Suzuki, Daisuke Ogawa, Takahiro Kanda, Takeshi Fujimori, Yuki Shibayama, Asadur Rahman, Juanjuan Ye, Hiroyuki Ohsaki, Kazuya Akimitsu, Ken Izumori, Takashi Tamiya, Akira Nishiyama, Keisuke Miyake

**Affiliations:** 1 Department of Neurological Surgery, Faculty of Medicine, Kagawa University, 1750-1 Ikenobe, Miki-cho, Kita-gun, Kagawa 761-0793 Japan; 2 Department of Pharmacology, Faculty of Medicine, Kagawa University, 1750-1 Ikenobe, Miki-cho, Kita-gun, Kagawa 761-0793 Japan; 3 Molecular Oncologic Pathology, Department of Pathology and Host-Defense, Faculty of Medicine, Kagawa University, 1750-1 Ikenobe, Miki-cho, Kita-gun, Kagawa 761-0793 Japan; 4 Department of Medical Biophysics, Kobe University Graduate School of Health Science, 7-10-2 Tomogaoka, Suma-ku, Kobe, 654-0142 Japan; 5 International Institute of Rare Sugar Research and Education, Kagawa University, 2393 Ikenobe, Miki-cho, Kita-gun, Kagawa 761-0795 Japan

**Keywords:** Cell biology, Cancer

## Abstract

Recent studies have shown that D-allose, a rare sugar, elicits antitumor effects on different types of solid cancers, such as hepatocellular carcinoma, non-small-cell lung cancer, and squamous cell carcinoma of the head and neck. In this study, we examined the effects of D-allose on the proliferation of human glioblastoma (GBM) cell lines (i.e., U251MG and U87MG) in vitro and in vivo and the underlying mechanisms. D-allose treatment inhibited the proliferation of U251MG and U87MG cells in a dose-dependent manner (3–50 mM). However, D-allose treatment did not affect cell cycles or apoptosis in these cells but significantly decreased the cell division frequency in both GBM cell lines. In a subcutaneous U87MG cell xenograft model, intraperitoneal injection of D-allose (100 mg/kg/day) significantly reduced the tumor volume in 28 days. These data indicate that D-allose-induced reduction in cell proliferation is associated with a subsequent decrease in the number of cell divisions, independent of cell-cycle arrest and apoptosis. Thus, D-allose could be an attractive additive to therapeutic strategies for GBM.

## Introduction

Glioblastoma (GBM) is one of the most devastating primary malignancies occurring in the central nervous system, accounting for approximately 15% of all tumors^1,2^. Because of its high invasiveness and the possibility of neurological deficits or deterioration after resection, radical resection is difficult in most GBM cases. The Stupp regimen, the standard radiochemotherapy strategy, extended the median survival to 15 months^3,4^, and subsequent therapies, such as bevacizumab, a molecular-targeting drug, and nivolumab, an immunotherapeutic agent, have been investigated but did not extend the overall survival^5–7^. Thus, developing novel therapeutics for GBM remains challenging.

Rare sugars are a general term for monosaccharides that exist only in very small quantities in nature, and approximately 50 types of rare sugars have been identified to date. D-allose, the epimer of D-glucose at position 3, has been reported to have several biological functions, such as antidiabetic and neuroprotective effects, in vivo^8–10^. Recently, several studies have reported the antitumor effects of D-allose. Sui et al*.*^11^ have reported that D-allose inhibits tumor growth in ovarian carcinoma cells. Yamaguchi et al*.*^12^ have shown that D-allose inhibits tumor growth in a dose-dependent manner in hepatocellular carcinoma cells through G1 arrest with the overexpression of the thioredoxin interacting protein gene and stabilization of p27^kip1^ (G1/S cycle transition regulatory protein). Furthermore, D-allose reduced the expression of glucose transporter-1 and decreased glucose uptake in hepatocellular carcinoma (HuH-7)^13^. In contrast, D-allose combined with cisplatin enhanced the efficacy of radiochemotherapy in a subcutaneous tumor model of non-small-cell lung cancer^14^. The enhanced efficacy and safety of radiochemotherapy with D-allose have also been reported in a subcutaneous tumor xenograft of squamous cell carcinoma of the head and neck^15^.

Importantly, no studies have reported the effects of D-allose on brain tumors, particularly gliomas. Furthermore, the anticancer mechanism of D-allose remains unclear because many results have been reported^11–15^. Therefore, this study examined the effects of D-allose on the growth of GBM cell lines, which have a poor prognosis among brain tumors. Our data have demonstrated for the first time that D-allose elicits antitumor effects on GBM cells by reducing the speed of cell division, independent of cell-cycle arrest and apoptosis.

## Results

### D-allose inhibits cell proliferation in GBM cells

**Figure 1:**
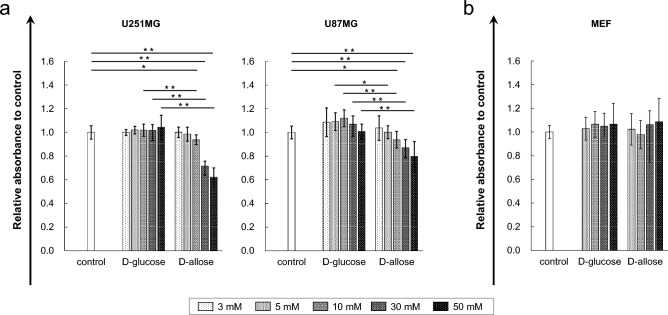
Antiproliferative effects of D-allose on human GBM cell lines and toxicity against healthy fibroblast cells in vitro. The graph shows the results of the WST-1assay of various D-allose treatments in GBM cell lines (**a**) and healthy fibroblast cells (**b**). Tests were performed on U251 (*n* = 12 in each concentration), U87MG (*n* = 12 in each concentration), and MEF cells (*n* = 12 in each concentration). Comparisons were also performed with D-glucose-treated samples at the same concentration. Statistically significant (*P* < 0.01, *P* < 0.001) reductions in cell proliferation compared with that in the control or D-glucose-treated samples are represented by asterisks (*, **). GBM, glioblastoma; WST-1, water-soluble tetrazolium-1; MEF, mouse embryonic fibroblast.

Cell proliferation was evaluated using the WST-1 assay in both U251MG and U87MG cells. The mean relative cell viability was 1.00 ± 0.06 for the control group, 0.94 ± 0.04 for 10-mM D-allose (*P* < 0.01, *n* = 12), 0.72 ± 0.08 for the 30-mM D-allose treatment group (*P* < 0.001, *n* = 12), and 0.62 ± 0.08 for the 50-mM D-allose treatment group (*P* < 0.001, *n* = 12) in the U251MG cell line, and it was 1.00 ± 0.06 for the control group, 0.94 ± 0.07 for the 10-mM D-allose treatment group (*P* < 0.01, *n* = 12), 0.87 ± 0.08 for the 30-mM D-allose treatment group (*P* < 0.001, *n* = 12), and 0.80 ± 0.13 for the 50-mM D-allose treatment group (*P* < 0.001, *n* = 12) in the U87MG cell line (Fig. 1a). These data revealed that D-allose treatment reduced the viability of U251MG and U87MG cells in a dose-dependent manner.

Considering the osmotic pressure, a comparison between the D-allose and equimolar D-glucose treatments showed a clear significant difference in both U251MG and U87MG (Fig. 1a). These data suggest that D-allose elicits antiproliferative effects independent of its osmotic pressure.

Moreover, toxicity was also evaluated using the WST-1 assay in Mouse embryonic fibroblast (MEF) cells. The mean relative cell viability was 1.00 ± 0.07 for the control group and 1.09 ± 0.20 for the 50-mM D-allose treatment group (*P* = 0.99, *n* = 12). A comparison between the D-allose and equimolar D-glucose treatments showed no significant difference. These data indicate that up to 50 mM D-allose has no toxicity in MEF cells (Fig. 1b).

### D-allose suppresses tumor growth in GBM xenograft mice

**Figure 2:**
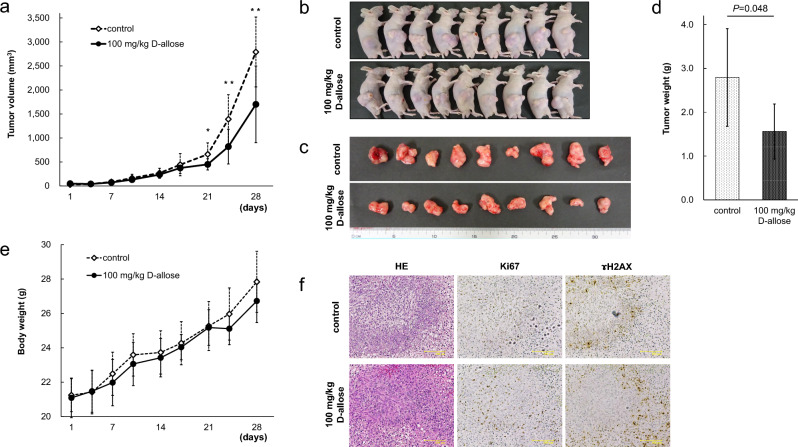
Antitumor effects of D-allose on human GBM cell lines in vivo. Eighteen subcutaneous tumor model mice of U87MG were divided into the control and D-allose groups. Each of them was administered with natural saline or D-allose solution intraperitoneally daily. (**a**) The graph shows tumor volume changes over time in each group. (**b**) A picture showing mice with tumors. (**c**) A picture showing resected tumors on the 28^th^ day. (**d**) The graph shows the weight of the resected tumors in each group. € The graph shows changes in body weight over time. (**f**) Representative images show HE and immunohistochemistry staining for Ki67 and ɤH2AX. Statistically significant (*P* < 0.05, *P* < 0.01) decreases in tumor volume compared with that in the control group are represented by asterisks (*, **)(Bar = 100 µm). GBM, glioblastoma; HE, hematoxylin and eosin.

We prepared 18 subcutaneously U87MG-injected xenograft mice and treated them with D-allose. After 21 days, a significant difference was observed between the two groups (control: 659 ± 238 mm^3^ vs. D-allose: 450 ± 117 mm^3^; *P* = 0.03, *n* = 9 for each group), and these differences gradually increased each day. The experiment was terminated when an obvious difference was observed in tumor growth on the 28th day (2791 ± 727 mm^3^ vs. 1700 ± 796 mm^3^; *P* = 8.0 × 10^−6^, *n* = 9 for each group) (Fig. 2a). The mice were anesthetized (Fig. 2b), and the resected tumors appeared smaller in the D-allose-treated group than in the control group (Fig. 2c). A significant difference in the weight of the resected tumors was observed (Fig. 2d). No mouse was eliminated because of excessive tumor growth. The body weight of the mice was very similar between the two groups (27.8 ± 1.8 g vs. 26.7 ± 1.3 g; *P* = 0.27, *n* = 9 for each group) (Fig. 2e). These results suggest that D-allose has antitumor effects on GBM in vivo. In contrast, no differences in the HE and immunohistochemically stained sections with anti-Ki67 and anti-ɤH2AX antibody were observed (Fig. 2f). This suggests that D-allose does not suppress the ratio of proliferating cells or affect DNA damage.

### D-allose has no observable effects on cell-cycle progression and apoptosis in GBM cells

**Figure 3:**
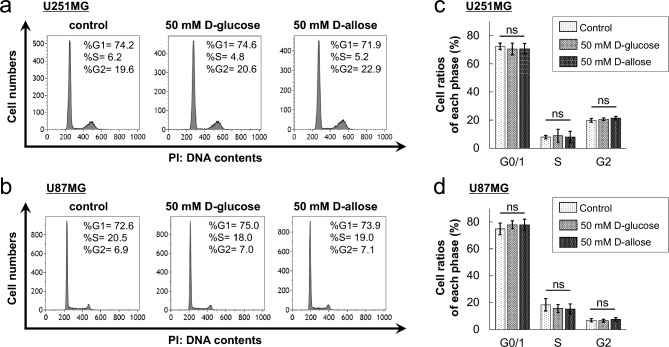
Cell-cycle modification of U251MG and U87MG cells. Cells were stained with PI, and the intensity of fluorescence was assessed at 617 nm. Representative data of the flow cytometry assay of U251MG (**a**) and U87MG (**b**) cells treated with control, D-glucose, or D-allose are shown. The cell population on the left convexity represents the G1 phase, that on the right represents the G2 phase. The values in each graph indicate the cell ratios of each phase. (**c**, **d**) The graphs show the mean values of four experiments for each group. PI, propidium iodide; ns, not significant.

U251MG and U87MG cells were cultured with or without 50-mM D-glucose or D-allose and were harvested, and their nuclei were stained with propidium iodide (PI). The percentage of cells in each cell-cycle phase was analyzed using flow cytometry. After 24 h of treatment, no observable difference was observed among the treatment groups (Fig. 3a, b). The proportions of cells in each phase were analyzed. In U251MG cells, no significant changes were observed in the proportion of cells in the G0/1 phase between the control (72.3% ± 2.2%), D-glucose (70.5% ± 4.2%; *P* = 0.40, *n* = 4), and D-allose treatment groups (70.7% ± 3.6%; *P* = 0.40, *n* = 4) (Fig. 3c). Conversely, in U87MG cells, the proportion tended to increase in D-allose-treated cells (77.9 ± 4.2%) compared with that in the control group (74.9% ± 4.4%) (*P* = 0.056, *n* = 4) (Fig. 3d). Because the proportion tended to increase in D-glucose-treated cells (78.0% ± 2.9%) (*P* = 0.067, *n* = 4), this tendency may not be unique to D-allose. Moreover, the results were similar when the treatment time was 96 h (data were not shown). These data suggest that D-allose did not affect the cell cycle in GBM cells.

**Figure 4:**
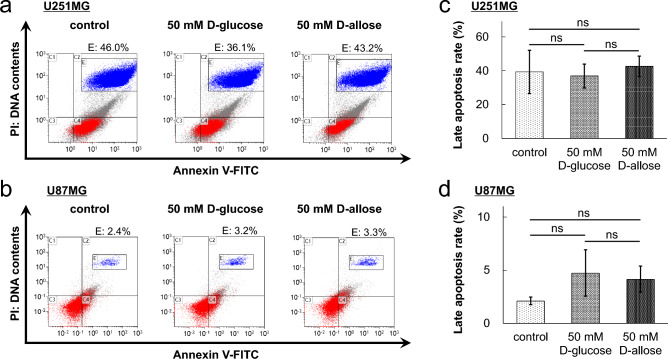
Apoptosis analysis of U251MG and U87MG cells. Cells were stained with FITC-labeled annexin V and PI, and the intensity of fluorescence was assessed at 525 nm (FITC) and 675 nm (PI). Representative data of the flow cytometry assay of U251MG (**a**) and U87MG (**b**) cells treated with control, D-glucose, or D-allose are shown. The values in each graph indicate the cell ratios of cells in late apoptosis. (**c**, **d**) The graphs show the mean values of three experiments for each group. FITC, fluorescein isothiocyanate; PI, propidium iodide; ns, not significant.

Flow cytometry was used to evaluate apoptosis in U251MG and U87MG cells. FITC-labeled annexin V and PI were used to identify apoptotic cells. In principle, cells in early apoptosis are positive for annexin V but negative for PI, and cells in late apoptosis or dead cells are positive for both annexin V and PI. Therefore, cells considered viable are negative for both annexin V and PI. In both GBM cell lines, no difference in the population of apoptotic cells was observed between cells treated with D-allose and those treated with equimolar D-glucose after 72 h (Fig. 4a, b). The proportion of cells in late apoptosis was analyzed. In U251MG cells, the mean proportion was 39.3% ± 12.8% for the control group, 36.9% ± 7.0% for the 50-mM D-glucose treatment group (*P* = 0.64, *n* = 3), and 42.7% ± 6.0% for the 50-mM D-allose treatment group (*P* = 0.52, *n* = 3), and no significant difference was observed between the groups (Fig. 4c). Conversely, in U87MG cells, the proportion tended to increase in the D-allose treatment group (4.2% ± 1.2%) compared with that in the control group (2.1% ± 0.4%) (*P* = 0.075, *n* = 4) (Fig. 4d). Furthermore, because the proportion also tended to increase in the 50-mM D-glucose treatment group (4.7% ± 2.2%) (*P* = 0.12, *n* = 4), this tendency may not be unique to D-allose. The results were similar when the treatment time was changed from 24 to 96 h, and therefore, we could not observe any apoptotic changes in GBM cells following D-allose treatment.

### D-allose reduces cell division frequency in GBM cells

Flow cytometry assay was performed to evaluate the cell division frequency in U251MG and U87MG cells using CytoTell™ Ultragreen. As stained cells divide, this reagent is equally distributed between daughter cells and can be measured as a successive halving of the fluorescence intensity of the dye. A difference in fluorescence intensity between any groups suggests that the cell division frequency differs between the groups; therefore, the higher the intensity, the lower the cell division frequency.

**Figure 5:**
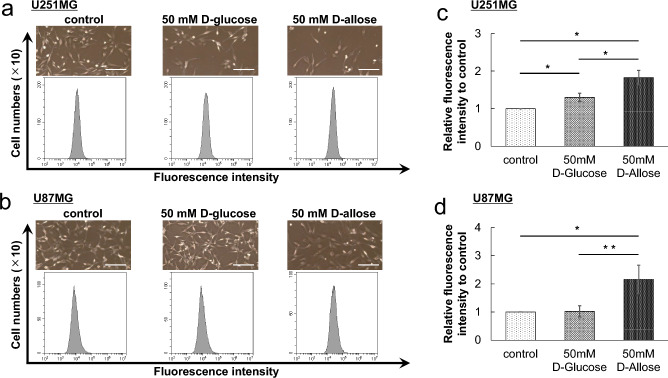
Cell division analysis of U251MG and U87MG cells. Cells were stained with CytoTell™ Ultragreen solution, and the intensity of fluorescence was assessed at 519 nm. Representative data of the flow cytometry assay of U251MG (**a**) and U87MG (**b**) cells treated with control, D-glucose, or D-allose are shown. Each photo was taken just before assay (Bar = 100 µm). The graphs show the mean value of the relative fluorescence intensity to the control of three (U251MG, **c**) or four (U87MG, **d**) experiments. Statistically significant (*P* < 0.05, *P* < 0.01) reduction in the cell division frequency compared with that in the control or D-glucose-treated samples is represented by asterisks (*, **). GBM, glioblastoma.

GBM cells were stained with CytoTell™ Ultragreen and cultured for 96 h to clarify these differences. Representative data showed that in D-allose-treated cells, the convexity of the graph shifted toward the higher side with differences in cell population appearance (Fig. 5a, b). These data have revealed that D-allose treatment significantly reduced the cell division frequency in U251MG cells (the mean relative fluorescence intensity was 1.00 for the control group, 1.30 ± 0.11 for the 50-mM D-glucose treatment group [*P* = 0.039, *n* = 3], and 1.82 ± 0.19 for the 50-mM D-allose treatment group [*P* = 0.017, *n* = 3]) and U87MG cells (the mean relative fluorescence intensity was 1.00 for the control group, 1.02 ± 0.20 for the 50-mM D-glucose treatment group [*P* = 0.86, *n* = 4], and 2.15 ± 0.51 for the 50-mM D-allose treatment group [*P* = 0.02, *n* = 4]) (Fig. 5c, d). Significant differences were observed between D-glucose- and D-allose-treated U251MG cells (*P* = 0.012) and U87MG cells (*P* = 0.0075).

## Discussion

Our in vitro and in vivo data revealed that D-allose treatment suppressed the proliferation of GBM cells. However, D-allose did not affect the cell cycle or apoptosis in these cells. Interestingly, D-allose treatment reduced the cell division frequency in GBM cell lines. Collectively, these data suggest that D-allose elicits antitumor effects through mechanisms independent of cell cycle arrest and apoptosis.

D-allose significantly inhibited cell proliferation in GBM cell lines (both U251MG and U87MG) in a dose-dependent manner. Similar concentration-dependent effects of D-allose were observed in head and neck squamous cell carcinoma^17^, ovarian cancer^11^, and hepatocellular carcinoma^12^ cells. Consistent with in vivo data with other cancers^14,15,17–19^, this study showed that GBM tumor growth was significantly inhibited by D-allose, which reduced the tumor volume to 60.9%. Thus, these data indicate that D-allose inhibits GBM cell proliferation. Moreover, the amount of D-allose administered intraperitoneally in this in vivo study, when converted to human body weight and glucose, is equivalent to 100 mL of 5% glucose solution once a day, which we thought would be easy to apply clinically.

Several mechanisms underlying the antitumor effects of D-allose have been reported. In ovarian cancer, Sui et al*.*^11^ observed that approximately 8% ± 3% of apoptotic cells were identified in cells treated with D-allose (50 mM/120 h). In human head and neck carcinoma, Mitani et al*.*^16^ observed moderate apoptosis induction in Ca9-22 cells treated with D-allose (50 mM for 72 h). Similarly, Indo et al*.*^17^ observed apoptosis in HSC3 cells elicited by D-allose treatment (25 mM for 5 days). Conversely, Yamaguchi et al*.*^20^ showed that D-allose (50 mM for 48 h) did not induce HuH-7 apoptosis, a hepatocellular carcinoma cell line. In this study, apoptosis was evaluated using flow cytometry; however, we did not observe any apoptotic changes in D-allose-treated cells. In U87MG cells, the proportion of cells in late apoptosis tended to increase compared with that in the control group; however, these changes were not statistically significant. Furthermore, the number of apoptotic cells tended to increase even in glucose-treated cells. Therefore, this tendency of D-allose is not unique and may be due to osmotic pressure or other effects. Thus, D-allose treatment (50 mM for 72 h) did not induce apoptosis in GBM cells. These data suggest that D-allose-induced cell apoptosis is not consistently induced in several cancer cell lines.

Several reports have revealed that D-allose induces cell-cycle arrest in several cancer cell lines. D-allose induces G1^12^ and S^16^ phase arrest, which was observed in head and neck cancer and hepatocellular carcinoma, respectively. Conversely, D-allose-induced G2/M arrest has been reported in head and neck cancer, non-small-cell lung cancer, and ovarian cancer^11,14,17^. However, no cell-cycle arrest was observed in D-allose-treated cells. In U87MG cells, the G0/1 phase tended to increase; however, this change was not statistically significant. These data combined with those of apoptosis support the notion that cellular responses (e.g., changes in cell cycles and induction of apoptosis) to D-allose are inconsistent in various cancer cells^16^. Moreover, the results of this study suggest that the antitumor effects of D-allose on GBM cells are mediated by an alternative mechanism.

To confirm the effects of D-allose on cell proliferation, the cell division frequency was investigated using the label-retaining method. Data showed that D-allose treatment significantly reduced the number of cell divisions compared with that in the control and D-glucose treatment groups. These data combined with those of other experiments indicated that D-allose decreases the number of cell divisions, which are not mediated by cell-cycle arrest or cell apoptosis. Based on these data, we speculate that D-allose uniformly decelerates the entire cell cycle but does not arrest the cells in any specific cell-cycle phase. The results obtained in vivo, that is, no changes in pathological findings despite a significant decrease in tumor volume, are consistent with this hypothesis. However, note that the effects of D-allose might be different from those on widely known “slow-cycling cells,” which means a population of cells with an innately slow cell cycle or a population that has slowed its cell cycle to survive an unexpected environment, characterized by chemoresistance or tumor relapse in oncology^21,22^. In these cells, cell proliferation inhibition is thought to be due to the prolonged non-cycling state of each cell^22^. Therefore, our observation of a slow cell cycle is slightly different from that of slow-cycling cells. Therefore, further careful and detailed studies should be conducted in the future.

This study is the first to examine the effects of rare sugars on brain tumors. The rare sugar D-allose was found to have an antitumor effect on tumors arising from the brain, which is one of the most sugar-consuming organs in the body. This study has several limitations. First, the molecular mechanism responsible for the antitumor effects of D-allose remains unclear. Second, the brain uptake of D-allose is lower than that of D-glucose in healthy mice but is higher in ischemia-disrupted brains than in healthy brains^23^. Therefore, whether D-allose can be disrupted into brain tumors remains unclear, and this study did not examine intra-tumor D-allose. Third, only the U87MG cell line was used in the in vivo study. We attempted to use the U251MG cell line in an in vivo study; however, these cells failed to grow in nude mice. Fourth, combination therapies with radiotherapy or chemotherapy have not been investigated. Future studies should be conducted to address these issues.

This study demonstrated that D-allose treatment reduces the proliferation of GBM cells. Our data also suggest that D-allose decelerates the cell cycle through a mechanism that is independent of cell apoptosis and cell-cycle arrest. Further studies are required to determine the detailed molecular mechanism responsible for the antitumor effects of D-allose on brain tumors.

## Methods

### Reagent

D-allose and D-glucose were kindly supplied by the Rare Sugars Research Center, Kagawa University, Kagawa, Japan. All sugars were dissolved at varied concentrations for each assay protocol in sterile Dulbecco’s Modified Eagle Medium (DMEM) (Thermo-Fisher Scientific) or natural saline (Otsuka Pharmaceutical Factory).

### Cell culture

Human GBM cell lines (U251MG and U87MG) were obtained from the American Type Culture Collection (ATCC). These cell lines were maintained in DMEM with 10% fetal bovine serum (FBS) (Sigma-Aldrich) at 37 °C under 5% CO_2_/95% air in a humidified incubator. MEF cells were also obtained from the ATCC and were maintained in DMEM with 10% FBS (Sigma-Aldrich) and 1% penicillin–streptomycin (Thermo-Fisher Scientific) at 37 °C under 5% CO_2_/95% air in a humidified incubator.

### Cell proliferation assay

The water-soluble tetrazolium (WST)-1 assay was used to assess the proliferation ability of both cell lines following the manufacturer’s instructions (Takara Bio).

Briefly, cells were seeded in a 24-well plate and incubated until confluence reached approximately 30–40%. The cells were serum starved for 24 h, the medium was removed, and a fresh medium containing variant concentrations (0, 3, 5, 10, 30, and 50 mM) of D-glucose or D-allose was added. After a subsequent 48-h incubation, 50-µL WST-1 reagent was added in addition to the 500-µL cell culture medium in each well, and the mixture was incubated for 2 h at 37 °C. Finally, absorbance was measured at 450 and 690 nm using a microplate reader (Corona Electric Co.). The value was obtained by subtracting the absorbance at 690 nm from that at 450 nm. D-glucose was used as a control for equivalent sugar osmotic pressure.

### Cell toxicity assay

The WST-1 assay was used to assess the toxicity of MEF cells following the manufacturer’s instructions (Takara Bio). Briefly, cells were seeded in a 24-well plate and incubated until confluence reached approximately 30%–40%. The cells were serum starved for 24 h, the medium was removed, and a fresh medium containing variant concentrations (0, 5, 10, 30, and 50 mM) of D-glucose or D-allose was added. After a subsequent 48-h incubation, the WST-1 assay was performed as in the cell proliferation assay.

### Subcutaneous GBM cell xenograft tumor model

Experimental protocols and animal care were performed according to the guidelines for the care and use of animals established by Kagawa University and complied with the ARRIVE guidelines. The Animal Experimentation Ethics Committee of Kagawa University approved the experimental protocols (approval number: 2020-18648-1). Five-week-old male BALB/c (nu/nu) nude mice weighing 20 g were purchased from Charles River Co. and maintained in specific pathogen-free animal facilities under a controlled temperature (24 ± 2 °C) and humidity (55 ± 5%) with a 12-h light–dark cycle. The mice were bred by providing standard chow and water ad libitum. U87MG cells were grown in 15-cm dishes, and after reaching confluency of 70–80%, the cells were trypsinized and centrifuged. After removing the supernatant, the pellets were diluted with phosphate-buffered saline (PBS) to a final concentration of 5 × 10^6^ cells/100 µL. The cells were subcutaneously injected into the right flank of the mice under anesthesia with isoflurane. When the tumor size reached approximately 150 mm^3^, the mice were randomly assigned to two groups, and treatment was initiated. The mice were administered D-allose (100 mg/kg) as previously described^14,15,17^ by intraperitoneally injecting 200 µL of saline daily for 30 days. Control mice were treated with equivolume intraperitoneal saline injection. Tumor growth was monitored by measuring the local tumor diameter at the implant site using a digital caliper twice a week until the end of the experiment. Body weight was also measured simultaneously. Tumor volumes were calculated using the following formula: length × width^2^ × 0.5. The experiment was terminated when a significant difference in tumor size was observed between the two groups or the tumor size was > 10% of the body weight. The tumors were surgically removed, and their weights were measured. Tissues were fixed in 10% neutral formalin solution (Wako), embedded in paraffin, and dissected into 4-µm-thick sections. The sections were stained with hematoxylin and eosin (HE) (Nacalai Tesque). Immunohistochemical staining was also performed using anti-Ki67 rabbit monoclonal antibody at 1/200 dilution (Abcam) and anti-ɤH2AX rabbit monoclonal antibody at 1/480 dilution (Cell Signaling Technology), according to the manufacturer’s instructions. The stained sections were observed using a light microscope (Eclipse Ci, Nikon).

### Cell-cycle analysis

The cell cycle was assessed using PI/RNase staining solution (Cell Signaling Technology) following the manufacturer’s instructions. Briefly, cells were cultured with DMEM containing equimolar (50 mM) D-glucose or D-allose for 24 h. Then, the cultured cells were collected by trypsinization, washed twice with PBS, and fixed in 70% ethanol overnight at − 20 °C. After washing with PBS, the cells were stained with 500-µL PI reagent for 30 min at room temperature under the dark condition. After washing with PBS, the cells were resolved in the exact volume of PBS. Flow cytometric analysis was performed at 617-nm emissions. Data were analyzed using Kaluza Analysis Software (ver. 1.2, Beckman Coulter) and are presented as percentage changes in cell distribution at each phase of the cell cycle^12^.

### Apoptosis assay

Cells were cultured with DMEM for 72 h, with no additional sugar (DMEM alone) and equimolar (50 mM) D-glucose or D-allose. The cells were detached using 0.05% trypsin-ethylenediaminetetraacetic acid and centrifuged at 800 rpm for 5 min. Then, the cells were washed twice with PBS and resuspended in 100-µL annexin V binding buffer (1 ×) following the manufacturer’s instructions (Nacalai Tesque, Inc.). After staining, the cells were maintained with fluorescein isothiocyanate (FITC)-labeled annexin V and PI working solution for 15 min at room temperature, and subsequently, a 400-µL annexin V binding buffer was added. Flow cytometry was performed by monitoring emissions at 518 nm (FITC) and 617 nm (PI)^24^.

### Assessment of the cell division frequency

Cell division frequency was assessed using the label-retaining method using CytoTell™ Ultragreen (ATT Bioquest Inc). Cells were collected and stained with a dye working solution (1×) for 30 min at room temperature under the dark condition following the manufacturer’s protocol. After an adequate reaction, the cells were washed with PBS and cultured for 96 h with 0.1% FBS containing DMEM without additional sugar (DMEM alone) and equimolar (50 mM) D-glucose or D-allose. Then, the cells were harvested by trypsinization and washed with PBS. Flow cytometric analysis was performed by monitoring emissions at 519 nm. Using the obtained graph, the average fluorescence intensity was calculated using CytExpert (ver. 2.3, Beckman Coulter).

### Statistical analysis

All data are expressed as means ± standard deviations. Differences in values between the control, D-glucose, and D-allose treatment groups were compared using *Student’s t*-test in both in vitro and in vivo studies. Statistical analyses were performed using EZR (Saitama Medical Center, Jichi Medical University), a graphical user interface for R (The R Foundation for Statistical Computing). *P-*values < 0.05 were used to denote statistical significance.

## Data Availability

The datasets analyzed during this study are available from the corresponding author on reasonable request.
